# The Conscious Limitation of the Offspring

**Published:** 1914-06

**Authors:** 


### The Conscious Limitation of the Offspring.

The conscious limitation of the offspring is one of the tenets of the eugenic propaganda. It opposes the old idea of unrestricted procreation which has been inherent in the human breast for so many centuries. Therefore it is not quietly accepted as gospel truth. However, many intelligent members of society have been deliberately practicing regulation for economical reasons By so doing they have not been actuated by any accepted moral code but solely from dire necessity. In reality these good people have been living up to the highest moral precept according to eugenic teachings, without realizing this fact. That such a practice will be generally accepted as our rule of action in the near future can be predicted with perfect confidence. "The mills of the gods grind slowly"; so does any evolution in the morals of society. But as surely as education is the leveler of society there will be evolved new ideas with a new adjustment of principles and customs. This evolution will be upward in the interest of a better race.

As an evidence of this trend of public opinion, we herewith produce an article from Dr. J. Rutgers, of the Hague, Holland, published in the May *Critic and Guide:*

"The kingdom of Holland, which in former centuries had fought itself free from the clerical government of Spain, is perhaps the only country in the world where freedom of speech and press is not a fiction. Apart from criminal and obscene publications, every opinion is admissible by law and by post. Limitation of offspring is as freely discussed as artificial fecundation. Conscious regulation of one's offspring in accordance with one's existing means for living and for education, in accordance also with individual wishes, and last, but not least, with the health and energy of the parents, is freely preached in Holland as a common sense precaution as a matter of course. So Holland is the best test of the results following when neomalthusianism is discussed freely and when the giving of information is not checked by law.

"Intelligent and well-to-do people adopted the principle at once; the elite of the laboring class soon followed. But then came the danger that the unfit would multiply without limit and the fit would not multiply in the same ratio. Therefore the Neomalthusian League in Holland now doubles its efforts in order to deal with this transitional condition, and for twelve years our league has been working chiefly among the mass of laborers, where the information is also welcome as soon as it is brought within their reach. These last years even the very poor women begin to implore our assistance to obtain the appliance gratuitously.

"It is our experience that information is asked, first, for maintaining the standard of life in order to give to the children a good education and all necessities of life; second, for sparing the health of the mother. Especially in the middle class, and among the better paid laboring people, education of children is now careful and the bringing up joyous, where formerly scarcity and anxiety reigned. Children are now a blessing, not a curse; they are welcome, or they are not born. Just as in former times I often noticed that death of infants was a relief and was acknowledged as such; so now parents are anxious for all that concerns the good health of the children. In this respect a reciprocal action may be observed: in families where children are carefully procreated they are reared carefully, and, where children are carefully treated, they are carefully procreated.

"So there are few countries where, proportional with the falling birth rate, the death rate of children in the first year of life has fallen so rapidly as in Holland, and our surplus of births over deaths is among the highest in the world, as Dr. C. V. Drysdale shows in his statistical diagrams.

"Indeed, parental prudence is no race suicide, as could be presumed a priori. The statistical figures in Holland that cannot be denied prove that in practice neomalthusianism is a factor of race improvement. We see it in every case. Rich people that are too lazy, too luxurious, too selfish to want children, will die without leaving offspring. Poor people that are too miserable will also refuse to have children, since the laws forbid wage-work for children. Every mother that feels herself weak, exhausted, suffering, will prevent procreation. Only individuals that feel themselves happy, efficient, energetic, in good health, individuals that feel endowed with a good humor and who love children heartily, only they will procreate, and that is all we want. It is conscious selection instead of brute natural selection. It is the same principle that all breeders of races in the animal kingdom and all gardeners have long since realized; there is no race improvement without limitation of numbers. Only in the human being it is the mother herself who is conscious if she feels well enough for this highest of all missions. How can anyone imagine that ignorance and carelessless should be more propitious for the future of the race than intelligent consciousness?

"The Neomalthusian League in Holland has worked now nearly thirty-three years in spreading knowledge. Mr. S. Van Houten, afterwards Prime Minister, having prepared public opinion since 1877, the league was founded by Mr. C. V. Gerritsen, a well known business man and statesman, in Amsterdam in 1881, only four years after the inauguration of the first Neomalthusian League in London, created by Dr. C. R. Drysdale, Sr.

"This was a revival of public consciousness in sexual matters against ignorance and obscurantism. The first woman doctor in Holland, Dr. Aletta H. Jacobs in Amsterdam, was the first doctor to give information gratis to poor women. Our headquarters at The Hague and our subdivisions in all our greater towns are spreading theoretical leaflets and pamphlets; but the special pamphlet giving practical information in the prevention of conception is only given to married people when asked. We are lecturing everywhere. But the essential missionary work is done privately and modestly, often unconsciously by showing the happy results in their own families, by the nearly 5000 members of our league spread over the whole country, among whom are physicians, clergymen and teachers, etc. Every day information is asked by letters and still more by our printed postcards; all information is given cost-free and post-free. Almost all younger doctors and midwives are giving information, and are helping mothers in the cases when it is wanted on account of pathological indications. Moreover, special nurses are instructed in helping poor women. Harmless preventive means are more and more taking the place of dangerous abortion. So, merely by our freedom of giving information, we have reached the desirable results proved most brilliantly by the statistical figures of our country.

"Certainly there are abuses, but the abuses of knowledge are never so numerous as the abuses of ignorance. And hygiene is the highest form of morality."

